# Racial disparities in acute care utilization among individuals with myasthenia gravis

**DOI:** 10.3389/fpubh.2025.1448803

**Published:** 2025-02-03

**Authors:** Cynthia Qi, Pushpa Narayanaswami, Ashley E. L. Anderson, Deborah Gelinas, Yuebing Li, Jeffrey T. Guptill, Dakshinamoorthy Amirthaganesan, Charlotte Ward, Rupesh Panchal, Amit Goyal, Glenn Phillips

**Affiliations:** ^1^Argenx US Inc., Boston, MA, United States; ^2^Department of Neurology, Beth Israel Deaconess Medical Center, Boston, MA, United States; ^3^Department of Neurology, Houston Methodist, Houston, TX, United States; ^4^Neuromuscular Center, Cleveland Clinic, Cleveland, OH, United States; ^5^ZS Associates, Bengaluru, Karnataka, India; ^6^ZS Associates, Bethesda, MD, United States; ^7^ZS Associates, Thousand Oaks, CA, United States; ^8^ZS Associates, Princeton, NJ, United States

**Keywords:** myasthenia gravis, racial disparities, social inequalities, social determinants of health, healthcare resource utilization, acute care, myasthenic exacerbation, race

## Abstract

**Objective:**

In myasthenia gravis (MG), evidence on the impact of social determinants of health on disparities in disease burden and healthcare resource utilization is limited. This study aimed to investigate the independent association between race/ethnicity and acute care utilization during the 2 years post-diagnosis among patients with MG.

**Methods:**

A retrospective cohort study was conducted among adults (≥18 years) with newly diagnosed MG in the United States using Optum’s de-identified Market Clarity Data from January 1, 2010, to December 31, 2019. Multivariable regression models were used to assess the association between acute care utilization and race/ethnicity, insurance, exacerbation at index, and other covariates.

**Results:**

A total of 7,058 patients met the study inclusion criteria, of whom 57% (*n* = 4,052) identified as Caucasian, 6% (*n* = 445) African American, 3% (*n* = 235) Hispanic, 1% (*n* = 94) Asian, and 32% (*n* = 2,232) with missing race/ethnicity information. Compared with patients identifying as Caucasian, those identifying as African American had 37% higher odds of having an emergency department visit in year 1, and those identifying as Hispanic had 70% increase in odds of having a hospitalization event in year 2 post-diagnosis. Among other covariates, Medicaid usage, exacerbation at index, and number of outpatient visits were significantly associated with acute care utilization.

**Conclusion:**

Racial disparities significantly impacted acute care utilization in the first 2 years post-MG diagnosis. Future studies should aim to examine specific factors that may contribute to disparities such as barriers to healthcare access, greater severity of MG symptoms, and poorly controlled disease.

## 1 Introduction

Myasthenia gravis (MG) is a chronic autoimmune neuromuscular disorder characterized by defective transmission at the junction between motor nerves and muscles (1). Currently, more than 60,000 individuals live with MG in the United States (US), with 80% having generalized MG symptoms including muscle fatigue with use that improves with rest, weakness that can affect mobility, cause eyelid droop and double vision, difficulties swallowing/chewing, and breathing dysfunction (1–5). Compared with healthy individuals, patients with MG are 2.6 times more likely to be hospitalized and 4.5 times more likely to be admitted to an intensive care unit (6). This is in large part due to the risk of acute complications and exacerbations of MG, including myasthenic crises, which are defined by the requirement for ventilatory support and management in intensive care (7). Myasthenic crisis can occur at an overall median of 8–12 months from disease onset and can be the initial presentation of MG in a small number of patients (7–9). Patients with MG have described living with anxiety and fear of acute complications (10–13), and economic data have shown the high impact of acute events on cost burden in MG (14–16). These data collectively demonstrate that acute care utilization is an important driver of clinical, humanistic, and economic burden in MG.

There is evidence in other disorders that social and economic inequalities contribute to burden of disease. For example, in chronic conditions such as diabetes, social determinants of health (SDOH) have been found to account for 50–60% of health outcomes, with patients identifying as African American or Hispanic and those with lower socioeconomic status being associated with higher rates of illness and death (17). While evidence linking racial disparities to outcomes is limited in MG, one study reported that among patients with MG who were hospitalized, those identifying as African American were significantly more likely to experience systemic infections, be intubated, and receive mechanical ventilation compared with those identifying as Caucasian (18, 19). Combined with additional evidence reporting negative effects of SDOH barriers on patients with MG through the diagnosis and treatment experience (10, 18, 20, 21), these preliminary findings underscore a potentially sizeable impact of health disparities in MG (22–24); however, evidence on how these disparities contribute to MG disease burden is limited. Identifying and addressing potential risk factors associated with increased burden in early disease management is important to improve outcomes, quality of care, and experiences for all patients with MG and especially for those who are vulnerable and underrepresented.

The objective of this study was to investigate the independent association between acute care utilization and race/ethnicity (primary outcome) and other covariates, including SDOH (exploratory outcomes), during the first 2 years post-diagnosis among patients with MG in the US.

## 2 Materials and methods

### 2.1 Study design and data source

This study utilized Optum’s de-identified Market Clarity Data (January 1, 2007–December 31, 2021), which includes data from linked electronic health record and administrative claims from multi-payer sources in the US (25). Optum’s de-identified Market Clarity Data (Market Clarity) is an integrated, multi-source medical claims, pharmacy claims, and electronic health records data set. Market Clarity links electronic health record data including lab results, vital signs and measurements, diagnoses, procedures, and information derived from unstructured clinical notes using natural language processing with historical, linked administrative claim data including pharmacy claims, physician claims, clinical information facility claims and medications prescribed and administered. Market Clarity is HIPAA-compliant, statistician-certified, and de-identified. International Classification of Disease-9 (ICD-9) and ICD-10 codes were used to identify diagnoses. National Drug Codes and Current Procedural Terminology® codes were used to identify pharmacotherapies, services, and procedures across outpatient and inpatient visits. Outpatient visits were identified using procedure codes that consisted of evaluation and management visits but excluded laboratory and radiological visits. No identifiable or protected health information was obtained for use in this study.

### 2.2 Study population

**Figure 1:**
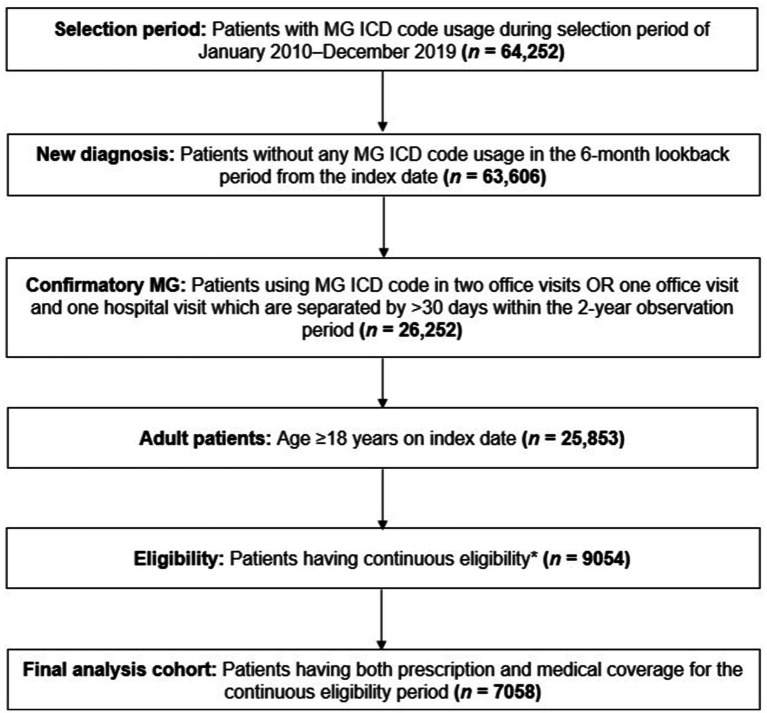
Patient selection funnel. *Continuous eligibility was defined as without any coverage gap >1 month starting from the 6-month lookback to the end of the 2-year observation period. ICD, International Classification of Disease; MG, myasthenia gravis.

Detailed inclusion criteria are summarized in Figure 1. Adult patients with new MG diagnosis spanning the period of January 1, 2010, to December 31, 2019, were identified using ICD-9 or ICD-10 diagnosis codes (358.0, 358.00, 358.01, G70.0, G70.00, G70.01). Each patient’s first MG diagnostic claim identified during the study period was used as the index date (26). We utilized a lookback period (period of time before index in which patients are required to not have any MG diagnostic claims) of 6 months in order to identify incident MG diagnoses. The 6-month lookback period was selected as a result of sensitivity analysis using 6-month, 1-year, and 2-year lookback periods. While approximately only 1% of patients were impacted by this variation in the confirmatory step due to the study period spanning 10 years, a larger impact was observed in the continuous eligibility step, with a larger proportion of patients being excluded with longer lookback periods. To retain sufficient patients to address the research question (including sub-cohort sizes), the 6-month lookback period was finally selected for this study.

Patients with confirmatory MG were defined as those with two office visits, or one office visit and one hospital visit linked to an MG diagnostic code, which were separated by ≥30 days and within the 2-year observation period (26). Further, patients were excluded if they had a medical or prescription insurance coverage gap greater than 1 month within the 2-year study period, as well as up to 6 months prior to their index date. Additional data regarding patient selection can be found in Supplementary Table 1.

### 2.3 Study variables and outcomes

Race/ethnicity (assessed at index date) was the primary study variable according to information and categories present in claims. Other study covariates included sex (female or male), age (continuous variable), and insurance status (commercial, Medicare, Medicaid, or multiple/unknown [included commercial and Medicaid; commercial and Medicare; Medicare and Medicaid; and commercial, Medicare, and Medicaid]), all assessed at index date.

Comorbidities (assessed during the 6 months prior to index date) included the following: Charlson Comorbidity Index (CCI) as a continuous variable (27) or categorial variable (<2 or ≥2, as sensitivity analysis), obesity/overweight (defined by ICD-9 or ICD-10 diagnosis codes present in the lookback period (28)), and baseline MG comorbidities (sleep disorder, anxiety, depression, autoimmune disorder, coronary artery disease, hypertension, hyperlipidemia, diabetes, thyroid disease, or gastroesophageal reflux disease [GERD]). Other MG-associated conditions included thymoma, which was assessed within the 2-year study period after index date. Exacerbation at index was defined as the presence of ICD diagnostic codes 358.01 or G70.01 (MG with [acute] exacerbation) at the index date. Patients whose MG diagnostic codes were all 358.01 or G70.01 throughout the observation period were not considered to have an exacerbation at index, due to likely coding error. Outpatient visits were defined as a continuous variable and measured by claims present during the observation period.

MG treatments used by patients with ≥1 treatment claim associated with an MG diagnosis were analyzed descriptively, including acetylcholinesterase inhibitors, glucocorticoids, nonsteroidal immunosuppressive treatments (NSISTs; including azathioprine, cyclophosphamide, cyclosporine, methotrexate, mycophenolate, and tacrolimus), intravenous immunoglobulin or subcutaneous immunoglobulin, plasma exchange, rituximab, and eculizumab. The reasons behind utilization of immunoglobulin and plasma exchange (e.g., maintenance therapy for disease control versus rescue therapy for intermittent exacerbations) were not available. Thymectomy was identified based on the presence of associated ICD-10-PCS (ICD-10 Procedure Coding System) codes (075M0ZZ, 075M3ZZ, 075M4ZZ, 0780–0784, 0795, 07BM0ZX, 07BM0ZZ, 07BM3ZX, 07BM3ZZ, 07BM4ZX, 07BM4ZZ, 07TM0ZZ, 07TM4ZZ).

The primary outcome was to measure the number of MG-related emergency department (ED) visit and hospitalization events during years 1 and 2 following MG diagnosis. As an explicit data field for primary diagnosis was unavailable in the dataset, we employed standard practice of considering diagnoses in the first 2 positions as a proxy. We defined visits as MG-related if the claim included an MG diagnosis in the first 2 positions. Hospitalizations were defined as MG-related if the most common diagnosis code (among positions 1 and 2) in the various claims during the hospital stay was MG. Outpatient visits were descriptively summarized. Additionally, among hospitalizations, crises, exacerbations, and thymectomy visits were descriptively summarized. Crisis-related hospitalizations were defined as the presence of one or more intubation claims with an associated inpatient stay and ICU (intensive care unit) admission. Exacerbation-related hospitalizations were defined by the presence of MG exacerbation ICD codes (G70.01or 358.01) with a concomitant absence of intubation claims. Thymectomy visits were defined as MG-specific inpatient hospital visits including a thymectomy ICD-10-PCS code. Since the primary focus of this study was to measure unplanned acute events in patients with MG, hospitalizations associated with thymectomy were excluded as these are usually elective procedures (29). Only MG-related acute care outcomes were considered in this analysis to avoid potential confounding from other comorbid conditions.

### 2.4 Statistical analysis

Descriptive statistics (e.g., mean, *N*, %) and bivariable analyses (Pearson chi-square tests for categorical variables and Student’s *t* tests for continuous variables) were used to compare patient characteristics across racial/ethnic subgroups. Bivariable analyses were also performed to assess whether patients with unknown/missing race/ethnicity data differed in their demographic and clinical characteristics compared with all those who had this information (as a single group; Supplementary Table 2).

To assess the primary outcome of measuring MG-related hospitalizations and ED visits, two independent multivariable logistic regression models focusing on each year were designed. For other acute care outcomes and treatment types, data are presented descriptively by racial/ethnic subgroups. Variables for inclusion in the model were determined by an evaluation of potential characteristics influencing acute care utilization in MG from the literature. Groupwise comparisons were calculated against all race categories. A *p*-value of <0.05 was considered statistically significant. All analyses were performed using Python 3.9.

## 3 Results

### 3.1 Study population demographics and characteristics

A total of 7,058 patients met the study inclusion criteria. Patients identifying as non-Hispanic Caucasian (hereafter referred to as Caucasian, 57%, [*n* = 4,052]) comprised a majority of the study population (Table 1).

**Table 1:** Baseline demographics and characteristics by racial/ethnic subgroup.

		Racial/ethnic subgroups					
	Overall	Non-Hispanic Caucasian	Non-Hispanic African American	Hispanic	Asian	Unknown	*p*-value^3^
*N* (%)	7,058	4,052 (57)	445 (6)	235 (3)	94 (1)	2,232 (32)	
Age, years							
Mean (SD)	61.93 (15.26)	63.56 (14.7)	52.72 (15.42)	56.5 (16.91)	56.43 (15.4)	61.62 (15.22)	<0.0001*
Sex, *n* (%)							
Female	3,644 (52)	2,015 (50)	294 (66)	142 (60)	55 (59)	1,138 (51)	<0.0001*
Male	3,413 (48)	2,036 (50)	151 (34)	93 (40)	39 (41)	1,094 (49)	
Insurance status, *n* (%)							
Commercial	3,322 (47)	1,920 (47)	209 (47)	112 (48)	52 (55)	1,029 (46)	<0.0001*
Medicare	2,349 (33)	1,450 (36)	93 (21)	72 (31)	24 (26)	710 (32)	
Medicaid	365 (5)	105 (3)	74 (17)	25 (11)	9 (10)	152 (7)	
Multiple/unknown^1^	1,022 (14)	577 (14)	69 (16)	26 (11)	9 (10)	341 (15)	
CCI							
Mean (SD)	1.33 (1.82)	1.33 (1.76)	1.25 (1.78)	1.38 (2.02)	1.13 (1.55)	1.37 (1.91)	0.71
0, *n* (%)	3,082 (44)	1,749 (43)	212 (48)	108 (46)	46 (49)	967 (43)
1–2, *n* (%)	2,708 (38)	1,574 (39)	155 (35)	85 (36)	34 (36)	860 (39)
3–4, *n* (%)	827 (12)	483 (12)	51 (11)	22 (9)	9 (10)	262 (12)
≥5, *n* (%)	441 (6)	246 (6)	27 (6)	20 (9)	5 (5)	143 (6)
Baseline MG comorbidities, *n* (%)^4^							
Hypertension	3,450 (49)	2,027 (50)	210 (47)	116 (49)	38 (40)	1,059 (47)	0.13
Hyperlipidemia	2,847 (40)	1,697 (42)	120 (27)	105 (45)	32 (34)	893 (40)	<0.0001*
Diabetes	1,866 (26)	1,051 (26)	116 (26)	66 (28)	21 (22)	612 (27)	0.59
Thyroid-related disorders	1,411 (20)	853 (21)	70 (16)	43 (18)	19 (20)	426 (19)	0.05
GERD	1,209 (17)	735 (18)	53 (12)	51 (22)	13 (14)	357 (16)	<0.0001*
Anxiety	827 (12)	508 (13)	49 (11)	33 (14)	11 (12)	226 (10)	0.05
Autoimmune	437 (6)	264 (7)	25 (6)	18 (8)	9 (10)	121 (5)	0.2
Depression	861 (12)	522 (13)	41 (9)	37 (16)	12 (13)	249 (11)	0.03*
Sleep disorder	984 (14)	596 (15)	48 (11)	38 (16)	14 (15)	288 (13)	0.07
Coronary artery disease	924 (13)	571 (14)	26 (6)	22 (9)	11 (12)	294 (13)	0.47
Other MG-associated conditions, *n* (%)^2^							
Thymoma	630 (9)	337 (8)	50 (11)	25 (11)	15 (16)	203 (9)	0.02*
Exacerbation diagnosis at index, *n* (%)	834 (12)	474 (12)	50 (11)	40 (17)	14 (15)	256 (11)	0.12

1 Includes commercial and Medicaid; commercial and Medicare; Medicare and Medicaid; and commercial, Medicare, and Medicaid.

2 Assessed within the 2-year study period after index date.

3 *p-values were calculated via the t-test for continuous variables and chi-square test for categorical variables. Groupwise comparisons were calculated against all race categories.*

4 Results for key common MG comorbidities of interest have been reported individually, regardless of whether the condition is included in the CCI calculation. Only the CCI was included as a variable in the models to avoid double counting.

*A *p*-value <0.05 was considered statistically significant.CCI, Charlson comorbidity index; GERD, gastroesophageal reflux disease; MG, myasthenia gravis, SD, standard deviation.

The mean (SD) age of the total study cohort was 61.93 (15.26) years. Patients identifying as African American were more than 10 years younger on average than patients identifying as Caucasian (mean, SD 52.72 [15.42] versus 63.56 [14.70] years, *p* < 0.0001). There were more females in the populations identifying as African American (66% [*n* = 294/445]) and Hispanic (60% [*n* = 142/235]) compared with Caucasians (50% [*n* = 2,015/4,052], *p* < 0.0001). Commercial plans and Medicare were the most common types of health insurance, with patients identifying as African American being the highest proportion of Medicaid users (17% [n = 74] versus overall mean, 5% [*n* = 365], *p* < 0.0001).

The proportion of patients identifying as African American had lower rates of hyperlipidemia (27% [*n* = 120] vs. 42% [*n* = 1,697], *p* < 0.001), GERD (12% [*n* = 53] vs. 18% [*n* = 735], *p* < 0.0001), and depression (9% [41] vs. 13% [*n* = 522], *p* < 0.0001) compared with patients identifying as Caucasian. Thymoma was significantly more common among patients identifying as Asian (16% [*n* = 15/94]) compared with patients identifying as Caucasian (8% [*n* = 337/4,052], *p* = 0.02) (Supplementary Table 3).

### 3.2 Treatment types and acute care utilization

The proportion of patients treated post-diagnosis with MG treatments decreased overall and across all cohorts from year 1 to year 2 (Supplementary Table 4). Treatment patterns across racial/ethnic subgroups were largely similar with some variations, including a trend of lower treatment utilization among patients identifying as African American (compared with other racial subgroups) in year 1. The frequency of thymectomy across racial/ethnic subgroups was generally similar in years 1 and 2.

Descriptive analyses of the utilization outcomes revealed that in both years 1 and 2, a higher proportion of patients identifying as African American (year 1: 16% [*n* = 73]; year 2: 10% [*n* = 43]) or Hispanic (year 1: 14% [*n* = 33]; year 2: 11% [*n* = 25]) had ED visits compared with patients identifying as Caucasian (year 1: 13% [*n* = 507]; year 2: 6% [*n* = 258]) or Asian (year 1: 5% [*n* = 5]; year 2: 5% [*n* = 5]; Supplementary Figure 1A). While patients identifying as African American had fewer hospitalizations in year 1 compared to other subgroups, patients identifying as Hispanic had more hospitalizations compared with other subgroups in year 2 (Supplementary Figure 1B). Patients identifying as African American (86% [*n* = 384]) or Hispanic (84% [*n* = 197]) had fewer outpatient visits compared with patients identifying as Caucasian (91% [*n* = 3,669]) or Asian (94% [*n* = 88]; Supplementary Figure 1C). Additional acute care outcomes were descriptively summarized (Supplementary Table 5).

### 3.3 Association between study covariates and acute care utilization

**Figure 2:**
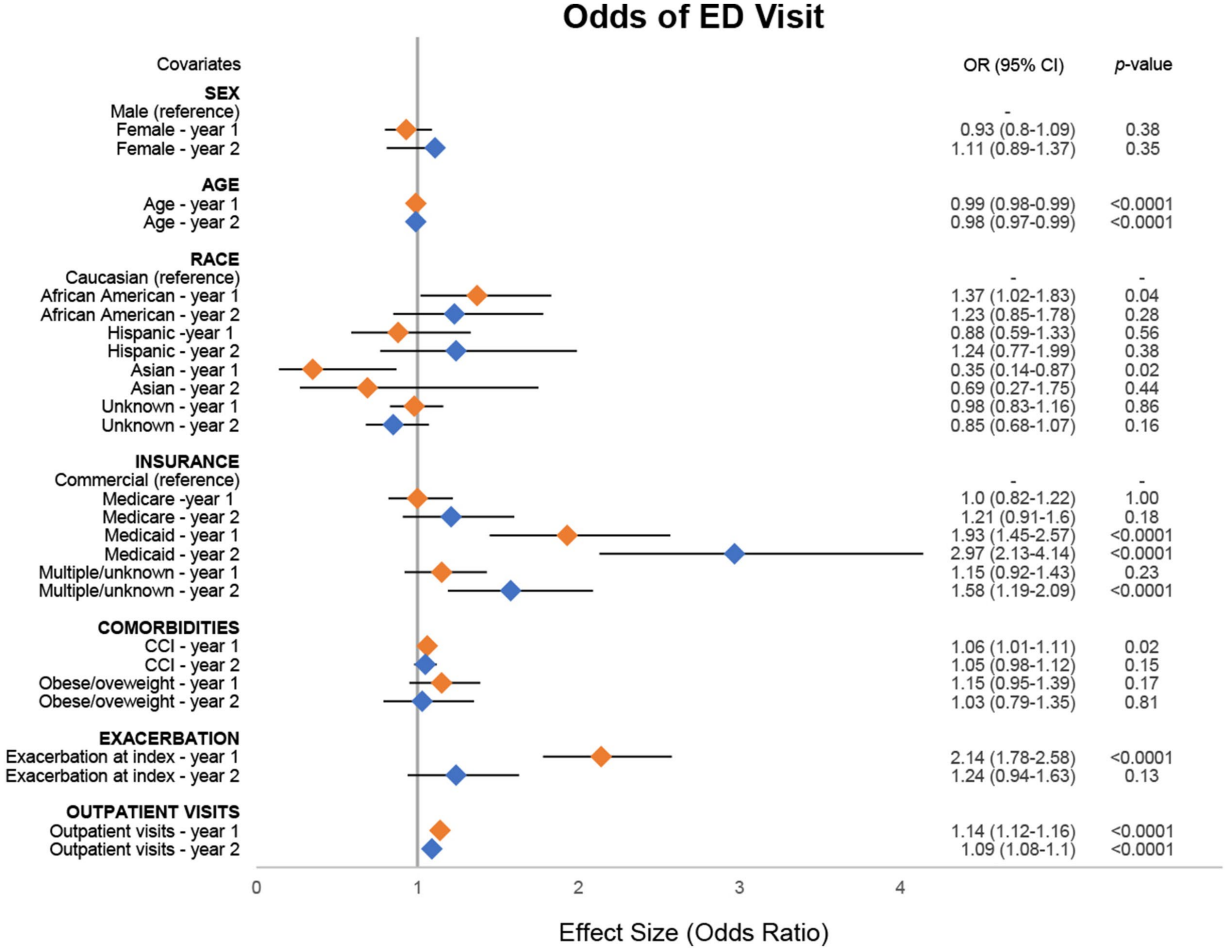
Odds of MG-related ED visit. Combined forest plot illustrating the separate multivariable regression model results for both year 1 (orange) and year 2 (blue). Odds ratios (ORs) correspond to the independent association between covariates and the odds of emergency department (ED) visit. ORs were calculated against the reference for categorical variables. Dots represent the OR, and the lines represent the 95% confidence interval (CI). A *p*-value of <0.05 was considered statistically significant. CCI, Charlson Comorbidity Index; MG, myasthenia gravis.

In adjusted analyses, we found several covariates assessed in this study had a significant association with ED visits (Figure 2; Supplementary Table 6). For every 1-year increase in baseline age of the patient, there was a corresponding 1% (95% CI: 1–2%) decrease in the odds of ED visits during year 1 and a 2% (95% CI: 1–3%) decrease in year 2. Patients identifying as African American had 37% (95% CI: 2–83%) higher odds of having an ED visit compared with those identifying as Caucasian in year 1. In contrast, patients identifying as Asian had 65% (95% CI: 13–86%) lower odds of having an ED visit compared with those identifying as Caucasian. Patients covered by Medicaid had 93% (95% CI: 45–157%) and 197% (95% CI: 113–314%) higher odds of having an ED visit in years 1 and 2 respectively, compared with patients using commercial insurance. There was a 6% (95% CI: 1–11%) increase in odds for every one-unit increase in CCI score, and those with an exacerbation at index had 114% (95% CI: 78–158%) higher odds of having an ED visit in year 1 compared with those without an exacerbation at index. For every one-unit increase in outpatient visits during the observation period, patients had corresponding 14% (95% CI: 12–16%) and 9% (95% CI: 8–10%) higher odds of experiencing an ED visit in years 1 and 2, respectively.

**Figure 3:**
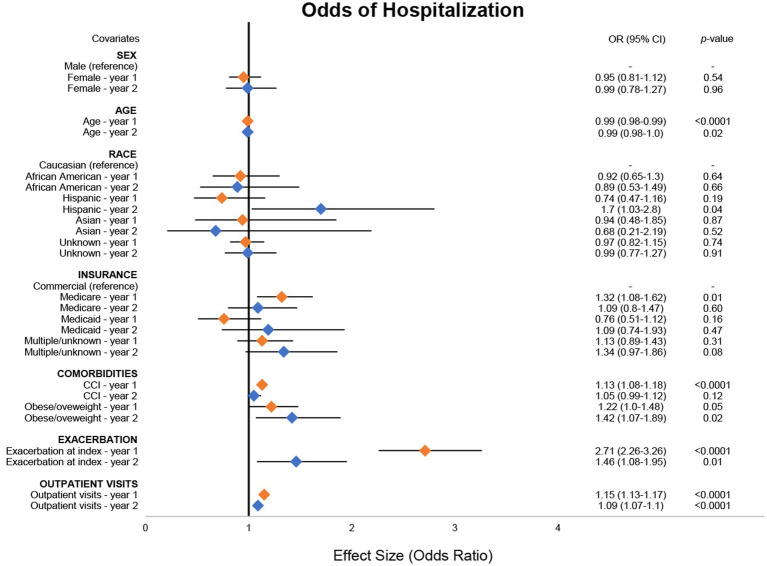
Odds of MG-related hospitalizations. Combined forest plot illustrating the separate multivariable regression model results for both year 1 (orange) and year 2 (blue). Odds ratios (ORs) correspond to the independent association between covariates and the odds of a hospitalization event. OR was calculated against the reference for categorical variables. Dots represent the OR, and the lines represent the 95% confidence interval (CI). A *p*-value of <0.05 was considered statistically significant. CCI, Charlson Comorbidity Index; MG, myasthenia gravis.

As was the case for ED visits, for every 1-year increase in age there was a corresponding 1% reduction in odds of hospitalization in both years 1 (95% CI: 1–2%) and 2 (95% CI: 1–2%), although estimates in year 2 were not statistically significant (Figure 3; Supplementary Table 7). In year 2, patients identifying as Hispanic had 70% (95% CI: 3–118%) higher odds of having a hospitalization event compared with patients identifying as Caucasian. Medicare users had 32% (95% CI: 8–62%) higher odds of having a hospitalization event compared with commercial insurance users in year 1. For every one-unit increase in CCI, there was a 13% (95% CI: 8–18%) increase in odds of a hospitalization. Patients who were obese/overweight at baseline had 22% (95% CI: 0–48%) and 42% (95% CI: 7–89%) higher odds of hospitalization in years 1 and 2, respectively, compared with patients who were not obese/overweight, although estimates in year 1 were not statistically significant. Patients with exacerbation at index had 170% (95% CI: 126–226%) and 40% (95% CI 8–95%) higher odds of having a hospitalization event in years 1 and 2, respectively, compared with those without an exacerbation at index. For every one-unit increase in outpatient visits during the observation period, there was a corresponding 15% (95% CI: 13–17%) and 9% (95% CI: 7–10%) increase in odds of hospitalization in years 1 and 2, respectively.

## 4 Discussion

This study investigated the association of race/ethnicity and other potential risk factors for increased acute care utilization during the first 2 years post-MG diagnosis. Our results further characterize both overall and health inequity-associated burden in MG, adding to previous evidence of the contribution of SDOH in health outcomes in MG (10, 14, 18, 20, 21).

### 4.1 Race/ethnicity impact

Our findings suggest that race/ethnicity can have a significant impact on early acute care utilization after MG diagnosis. Based on multivariable regression modeling, patients identifying as African American had higher odds of experiencing an ED visit compared with patients identifying as Caucasian in year 1. Although differences were not statistically significant for patients identifying as Hispanic across years 1 and 2 for ED visits, a higher proportion of them utilized ED visits compared with patients identifying as Caucasian, descriptively. These findings are consistent with results from the Medical Expenditure Panel Survey (2006–2013), which found that patients identifying as African American or Hispanic who had neurologic conditions were more likely to be cared for in the ED, and less likely to see an outpatient neurologist, relative to those identifying as Caucasian (30, 31). Evidence from other neurological conditions, such as Parkinson’s disease, have reported similar results, suggesting that patients identifying as African American or Hispanic may avoid outpatient care (due to negative historical experience or cultural beliefs/pressure) (31–35) or have barriers to regular outpatient visits after diagnosis, consequently using ED visits as surrogate for a visit (36).

There is also evidence that patients identifying as African American and or Hispanic may experience some symptoms of MG (e.g., ocular) at an earlier age, have more severe symptoms, and have refractory MG more commonly compared with patients identifying as Caucasian, which may also drive ED utilization, although additional research is needed (37–41). The mean (SD) age of the total study cohort was consistent with other US claims-based reports (18, 42, 43). Despite the mean baseline CCI being similar across racial/ethnic subgroups in our study, patients identifying as African American were more than 10 years younger in age on average compared with patients identifying as Caucasian, suggesting patients identifying as African American may experience earlier disease onset with additional comorbidity burden. A previous study based on national Veteran Affairs data found that patients identifying as African American or Hispanic had a 22 and 44% increased risk of exacerbation at index compared to those identifying as Caucasian, respectively (41). These findings illustrate that patients identifying as African American or Hispanic can face a complex set of barriers following MG diagnosis that may include clinical, economic, and cultural reasons, and may have challenges in managing the healthcare system with potentially limited support for health decision-making. Considering that acute care usage also poses additional burden on payers and healthcare institutions, further research should focus on the key drivers of unmet needs of patients with MG who may need additional support.

### 4.2 Socioeconomic impact

Our results showed that patients using Medicaid were nearly two- and threefold more likely to have an ED visit compared with commercial insurance users in years 1 and 2, respectively. These results highlight the impact of socioeconomic barriers in MG management through diagnosis, access, and treatment that have been previously reported across multiple studies (10, 14, 18, 20, 21). For example, in a study of 38 patients living with SDOH barriers and MG, one in three patients felt they received unequal treatment due to their socioeconomic status or avoided buying medication or doctor visits to save money (10). Moreover, patients using Medicaid expressed that insurance did not cover certain treatments, leading them to pay out of pocket for uncovered expenses (14). It is known that up to half of the patients with MG globally experience unemployment and reductions in income (44–47) and that low income is associated with a lower health-related quality of life (45), and acute care utilization is a major cost driver (14). Therefore, it becomes clear that socioeconomic barriers can substantially impact MG management and outcomes. Further evidence elucidating additional challenges such as inadequate or limited access to disease education, MG specialists, support teams, and treatment centers, which may cumulatively contribute to additional burden, should be generated to better support patients living with these barriers.

### 4.3 Impact of poorly controlled disease

In addition to race/ethnicity and insurance-specific risk factors, other attributes such as comorbidity burden, exacerbation at index, and increase in outpatient visits were also observed to be significantly associated with increased risk of acute care utilization. Holistically, these results suggest poorly controlled disease as a collective driver of acute care usage in the first 2 years after diagnosis. This time frame corresponds to when patients with MG are at the highest risk, as it has been reported that acute care utilization was the highest during year 1 in a separate US claims-based study, with 43% of patients with MG visiting the ED at least once (39). Similarly, most myasthenic crises, exacerbations, and MG-related hospitalizations occurred within 2–3 years of diagnosis in previous studies (6, 48, 49).

Our results suggest that specific factors contributing to poorly controlled disease exacerbate the risk. CCI and obesity are attributes that point to increased comorbidity burden, which often leads to poorer outcomes in MG (50, 51). Exacerbation at index suggests that patients may not have had received the appropriate access, management, or disease education, or that the disease is inherently more severe. While having outpatient visits can be reflective of adequate management, we observed an increase in the number of outpatient visits to be significantly associated with ED visits and hospitalizations. In this case, outpatient visits may be considered a surrogate for poorly controlled disease, as patients with MG require time to respond to certain treatments (and combinations), requiring several outpatient visits to a neurologist to find an appropriate treatment plan (39). The general decrease in utilization of MG treatments from year 1 to year 2 that we observed may be due to better disease control, although further research is necessary.

While our study focused on the first 2 years following MG diagnosis when patients are at high risk of exacerbations and crises, these events also greatly affect patients with MG beyond this time frame. A study based on national Veteran Affairs data demonstrated that the cumulative incidence of MG exacerbations increased from 11% at diagnosis to 45% at 15 years post-diagnosis (41). In several studies, patients with MG have reported the fear of experiencing exacerbations having a large impact on their life (10, 14). Given these previous studies and our results, it is clear that patients with poorly controlled disease have an unmet need that should be addressed.

### 4.4 Improving early MG management

Given our study findings, which illustrate patients identifying as African American or Hispanic have a greater risk of acute care utilization early after diagnosis, improving early disease management should be a primary focus in MG. Although direct evidence in MG is limited, better management early in the disease course is associated with improved outcomes in patients with neurological autoimmune conditions. In multiple sclerosis, for example, early treatment was associated with lower productivity losses, lower risk of reaching moderate disability, and better patient-reported physical symptoms compared with patients who had delays in treatment (52–55). Patient support programs, which can include medication management and patient education/counseling, can also bridge gaps in care and have been shown to increase medication adherence, reduce costs, and reduce healthcare utilization (56, 57). Patients living with SDOH barriers and MG have expressed needing more support at and after diagnosis. This effort requires increased awareness not only among neurologists but also primary care providers, nurses, opticians, ophthalmologists, and physical therapists, as well as patients and caregivers, to improve MG care management. In addition, further research is critical to address the potential impact of better management strategies and increased patient support at and early after diagnosis on both short- and long-term outcomes in MG.

### 4.5 Limitations

Administrative claims are subject to inherent inconsistencies in diagnostic and procedural coding practices. While the risk of missing data was minimized by ensuring a patient cohort with continuous activity in a closed claims dataset, the cohort was limited to that drawn from Optum’s de-identified Market Clarity Data provider network, which may not be fully representative of the entire US population especially when considering social determinants of health (i.e., demographics, healthcare access, insurance types, etc.). Consistent with previous US claims-based studies (18, 58), a large proportion of patients captured in this study identified as Caucasian or did not report their race/ethnicity, limiting cohort sizes for other racial/ethnic subgroups. Moreover, claims data systematically exclude uninsured patients, which historically includes many patients of color. There was limited literature and data available to further parse the impact of racial disparities in MG diagnosis rates and how it compares with healthcare resource utilization, warranting further studies in this area. Better SDOH data capture in claims, novel methods of alternative data collection, improved ways to engage people of color in research, and further studies are all needed to capture a more holistic picture of the impact of SDOH barriers and other societal risk factors on acute care utilization in MG. Due to limitations associated with administrative claims data, disease characteristics such as symptoms and severity of MG were not available to include in statistical modeling. However, exacerbation at index and number of outpatient visits were used as proxy measures to characterize disease severity based on evidence available.

While expected to contribute to acute care outcomes, MG treatments were not included as variables in the model for several reasons. First, descriptive results indicated variation in the utilization of different MG treatments between racial and ethnic subgroups, indicating a potential association. However, fully assessing what drives these differences requires richer data (i.e., disease duration, severity of symptoms, socioeconomic status, etc.) and more nuanced modeling techniques that capture the relationship among treatments and various other characteristics that influence treatment. It may not be the treatments themselves that necessarily impact acute care outcomes, but instead, other factors, which contribute to the receipt of that treatment. Second, MG treatment regimens and combinations are highly heterogenous across multiple aspects including treatment duration, combinations used, frequency, dosage levels, and fluctuations in dosage. A comprehensive analysis capturing these nuances leading up to the outcome requires alternative modeling techniques. Nevertheless, we recognize that the impact of MG treatments on acute care outcomes is of high interest and this should be evaluated in future studies that employ a causal modeling framework and longitudinal analysis to better inform additional drivers of acute care utilization among patients with MG. Finally, as the results we observed were based on statistical modeling only, the extent of impact on clinical outcomes needs to be further explored. Future studies with increasingly robust sample sizes and information regarding other variables such as disease severity and detailed treatment information should be leveraged to interrogate other attributes that could influence acute care utilization in MG.

## 5 Conclusion

Our study aimed to investigate the independent association between race/ethnicity and other study covariates and MG-related ED visit and hospitalization events during years 1 and 2 following MG diagnosis. By utilizing a multivariable regression model based on retrospective claims data, our study found that patients identifying as African American or Hispanic experienced significantly greater healthcare burden compared with those identifying as Caucasian. Moreover, other study covariates such as Medicaid insurance status, comorbidities, exacerbation at initial diagnosis, and increase in outpatient visits also contributed to greater acute care utilization. While additional research is needed to confirm the overall impact of SDOH, increased awareness and early disease management is pivotal in addressing gaps in care for patients with MG.

## Data Availability

The original contributions presented in the study are included in the article/Supplementary material, further inquiries can be directed to the corresponding author.
