# Supplementary material for: Racial disparities in acute care utilization among individuals with myasthenia gravis
Source: Front Public Health. 2025 Feb 3;13:1448803. doi: 10.3389/fpubh.2025.1448803 (PMC11832025; doi:10.3389/fpubh.2025.1448803)
Supplement: Supplementary file 1 [file Data_Sheet_1.docx]

Supplementary Material

Racial Disparities in Acute Care Utilization Among Individuals with Myasthenia Gravis

Cynthia Qi^*^, Pushpa Narayanaswami, Ashley E.L. Anderson, Deborah Gelinas, Yuebing Li, Jeffrey T. Guptill, Dakshinamoorthy Amirthaganesan, Charlotte Ward, Rupesh Panchal, Amit Goyal, Glenn Phillips

***Correspondence:** Cynthia Qi [cqi@argenx.com](mailto:cqi@argenx.com)

[Supplementary Figure 1. Acute care utilization by racial/ethnic subgroup. 2](#_Toc185430711)

[Supplementary Table 1. Inclusion criteria and screening parameters for individuals with MG. 4](#_Toc185430712)

[Supplementary Table 2. Comparison of race versus unknown group. 5](#_Toc185430713)

[Supplementary Table 3. Baseline demographics and characteristics of patients living with MG. 9](#_Toc185430714)

[Supplementary Table 4. Treatment types. 11](#_Toc185430715)

[Supplementary Table 5. Acute care outcomes. 13](#_Toc185430716)

[Supplementary Table 6. Odds of MG-related ED visit: Sensitivity analysis using CCI as a dichotomous variable. 16](#_Toc185430717)

[Supplementary Table 7. Odds of MG-related hospitalization: Sensitivity analysis using CCI as a dichotomous variable. 17](#_Toc185430718)

## Supplementary Figure 1. Acute care utilization by racial/ethnic subgroup.

1. **MG-related ED visits**


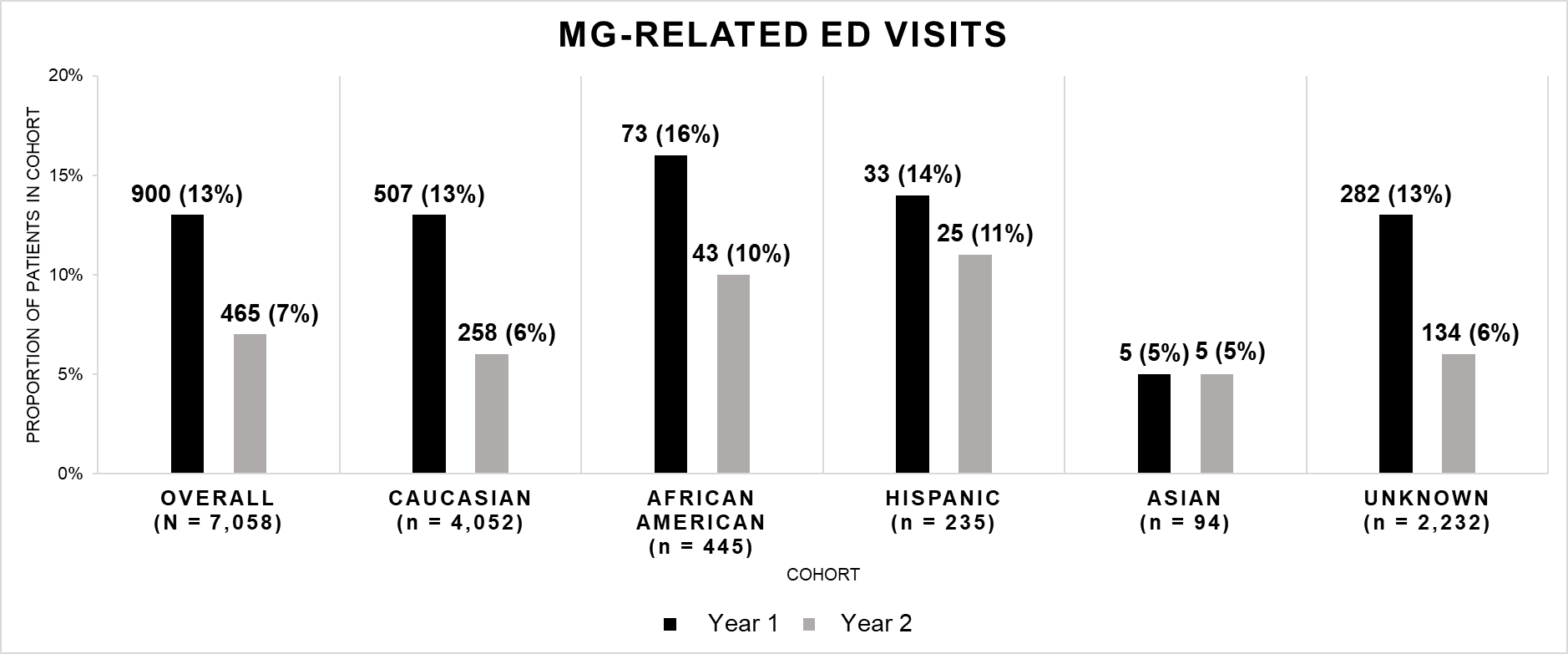


1. **MG-related hospitalizations**

**
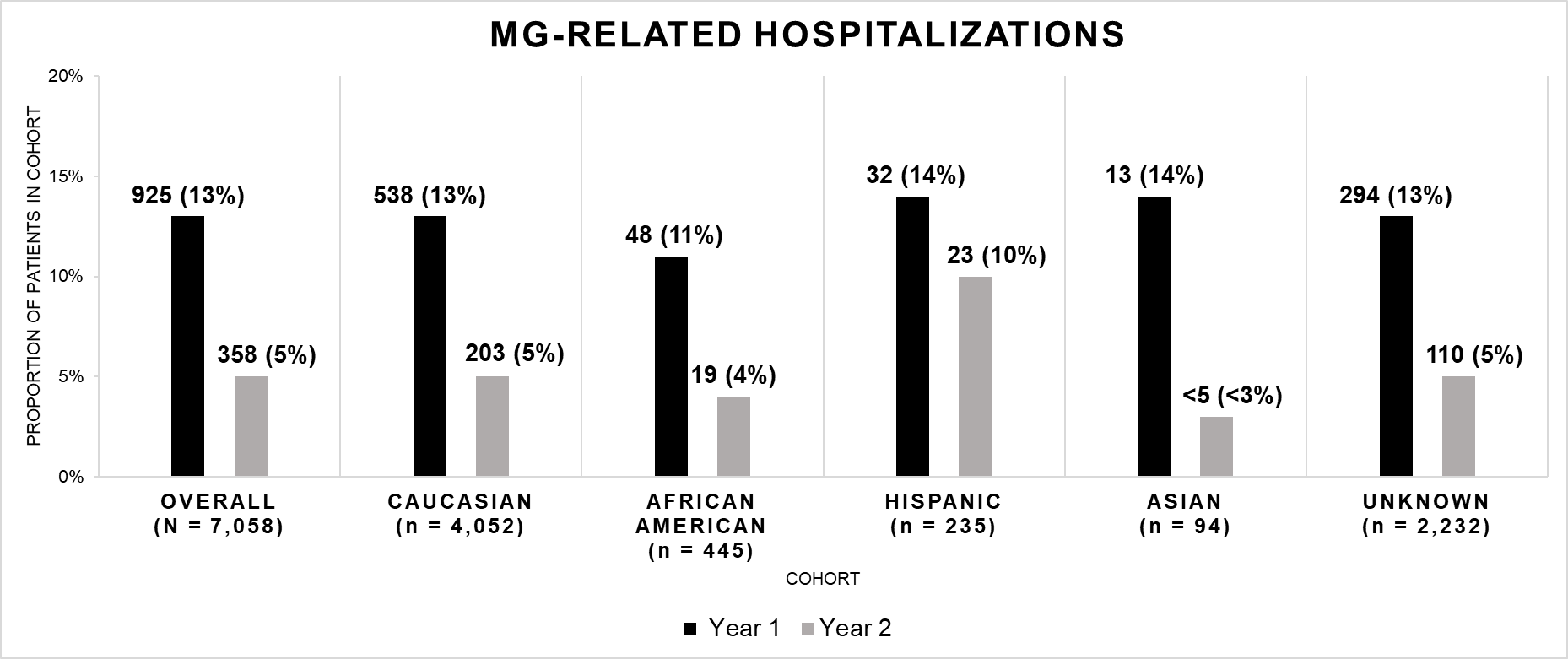
**

1. **Outpatient visits**


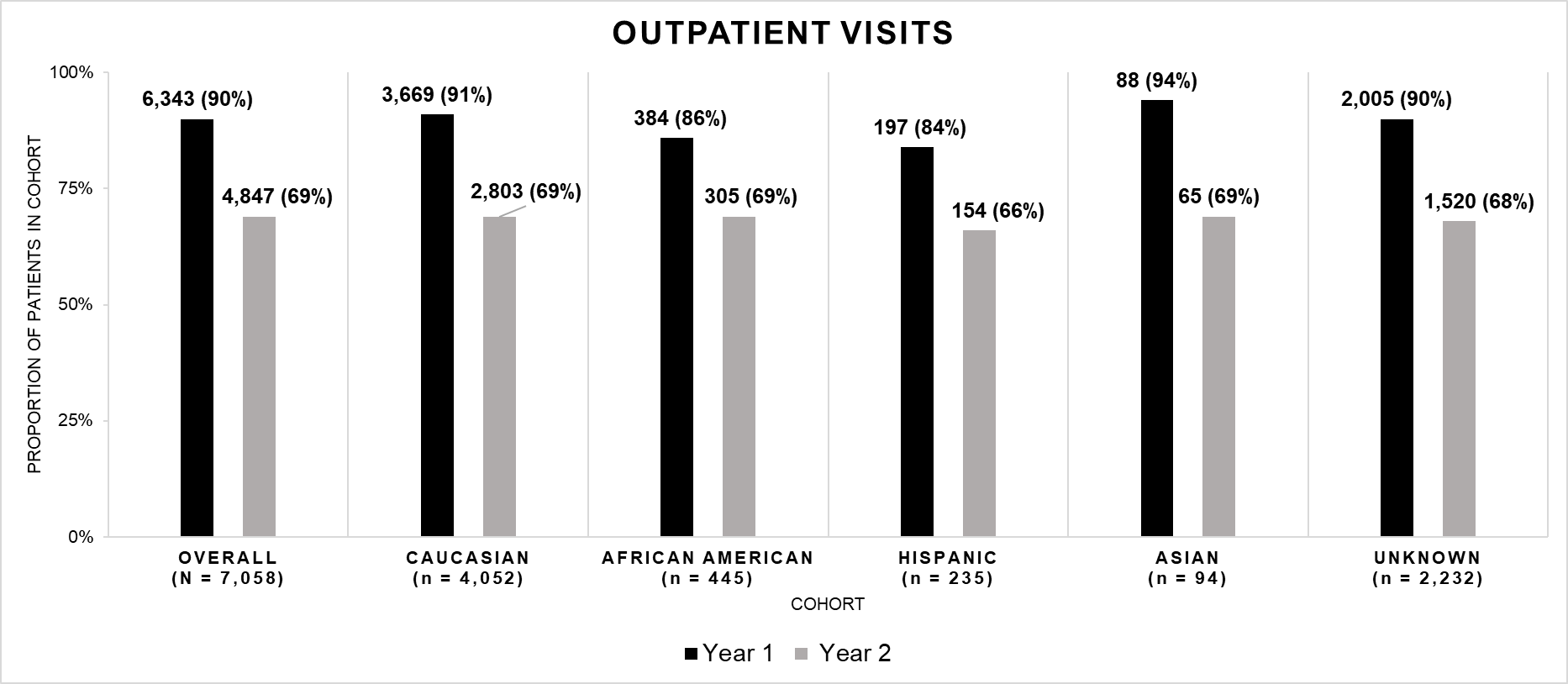


## Supplementary Table 1. Inclusion criteria and screening parameters for individuals with MG.

| **Patient Funnel** | **N** | **%** | **% drop from previous step** |
| --- | --- | --- | --- |
| **Selection Period:** Patients with MG ICD code^1^ usage during selection period of January 2010 to December 2019 | 64,252 | 100.00% | 0.00% |
| **New Diagnosis:** Patients without any MG ICD code usage within the 6-month lookback period from index date | 63,606 | 98.99% | 1.01% |
| **Confirmatory MG:** Patients using MG ICD code in two office visits OR one office visit and one hospital visit which are separated by >30 days and within the 2-year observation period | 26,252 | 40.86% | 58.73% |
| **Adult Patients:** Age ≥ 18 on index date | 25,823 | 40.19% | 1.63% |
| **Eligibility:** Patients having continuous eligibility, without any coverage gap greater than 1 month, starting from the 6-month lookback to the end of 2-year observation period for the patient (–6 months to +2 years with respect to index date) | 9,054 | 14.09% | 64.94% |
| **Final Analysis Cohort:** Patients having both prescription and medical coverage for the continuous eligibility period | 7,058 | 10.98% | 22.05% |

^1^MG ICD diagnosis codes: 358.0, 358.00, 358.01, G70.0, G70.00, G70.01.

## Supplementary Table 2. Comparison of race versus unknown group.

| **Age** | **All others** | **Unknown** |  |  | **Test values** |  |
| --- | --- | --- | --- | --- | --- | --- |
| Mean | 62.07853295 | 61.62007168 |  |  |  |  |
| Variance | 233.6048158 | 231.6117529 |  |  | *p*-value | 0.24 |
|  |  |  |  |  |  |  |
| **Sex** | **All others** | **Unknown** | **Row totals** |  | **Test values** |  |
| Female | 2,506 | 1,138 | 3,644 |  | Chi square stat | 0.55 |
| Male | 2,319 | 1,094 | 3,413 |  | Critical value | 3.84 |
| Column totals | 4,825 | 2,232 | 7,057 |  | *p*-value | 0.46 |
|  |  |  |  |  |  |  |
| **Region** | **All others** | **Unknown** | **Row totals** |  | **Test values** |  |
| Midwest | 2,053 | 576 | 2,629 |  | Chi square stat | 380.54 |
| Northeast | 878 | 816 | 1,694 |  | Critical value | 9.49 |
| Other/unknown | 174 | 118 | 292 |  | *p*-value | <0.0001 |
| South | 1,349 | 481 | 1,830 |  |  |  |
| West | 372 | 241 | 613 |  |  |  |
| Column totals | 4,826 | 2,232 | 7,058 |  |  |  |
|  |  |  |  |  |  |  |
| **Insurance** | **All others** | **Unknown** | **Row totals** |  | **Test values** |  |
| Commercial | 2,293 | 1,029 | 3,322 |  | Chi square stat | 21.15 |
| Medicare | 1,639 | 710 | 2,349 |  | Critical value | 7.81 |
| Medicaid | 213 | 152 | 365 |  | *p*-value | <0.0001 |
| Multiple/unknown^1^ | 681 | 341 | 1,022 |  |  |  |
| Column totals | 4,826 | 2,232 | 7,058 |  |  |  |
|  |  |  |  |  |  |  |
| **CCI** | **All others** | **Unknown** | **Row totals** |  | **Test values** |  |
| 0 | 2,115 | 967 | 3,082 |  | Chi square stat | 0.24 |
| 1-2 | 1,848 | 860 | 2,708 |  | Critical value | 7.81 |
| 3-4 | 565 | 262 | 827 |  | *p*-value | 0.97 |
| ≥5 | 298 | 143 | 441 |  |  |  |
| Column totals | 4,826 | 2,232 | 7,058 |  |  |  |
|  |  |  |  |  |  |  |
| **Comorbidities** | | | | | | |
|  | **All others** | **Unknown** | **Row totals** |  | **Test values** |  |
| **Hypertension** | 2,391 | 1,059 | 3,450 |  | Chi square stat | 2.69 |
| N/A | 2,435 | 1,173 | 3,608 |  | Critical value | 3.84 |
| Column totals | 4,826 | 2,232 | 7,058 |  | *p*-value | 0.10 |
|  |  |  |  |  |  |  |
|  | **All others** | **Unknown** | **Row totals** |  | **Test values** |  |
| **Hyperlipidemia** | 1,954 | 893 | 2,847 |  | Chi square stat | 0.15 |
| N/A | 2,872 | 1,339 | 4,211 |  | Critical value | 3.84 |
| Column totals | 4,826 | 2,232 | 7,058 |  | *p*-value | 0.70 |
|  |  |  |  |  |  |  |
|  | **All others** | **Unknown** | **Row totals** |  | **Test values** |  |
| **Diabetes** | 1,254 | 612 | 1,866 |  | Chi square stat | 1.62 |
| N/A | 3,572 | 1,620 | 5,192 |  | Critical value | 3.84 |
| Column totals | 4,826 | 2,232 | 7,058 |  | *p*-value | 0.20 |
|  |  |  |  |  |  |  |
|  | **All others** | **Unknown** | **Row totals** |  | **Test values** |  |
| **Thyroid-related disorders** | 985 | 426 | 1,411 |  | Chi square stat | 1.67 |
| N/A | 3,841 | 1,806 | 5,647 |  | Critical value | 3.84 |
| Column totals | 4,826 | 2,232 | 7,058 |  | *p*-value | 0.20 |
|  |  |  |  |  |  |  |
|  | **All others** | **Unknown** | **Row totals** |  | **Test values** |  |
| **GERD** | 852 | 357 | 1,209 |  | Chi square stat | 2.96 |
| N/A | 3,974 | 1,875 | 5,849 |  | Critical value | 3.84 |
| Column totals | 4,826 | 2,232 | 7,058 |  | *p*-value | 0.09 |
|  |  |  |  |  |  |  |
|  | **All others** | **Unknown** | **Row totals** |  | **Test values** |  |
| **Anxiety** | 601 | 226 | 827 |  | Chi square stat | 8.00 |
| N/A | 4,225 | 2,006 | 6,231 |  | Critical value | 3.84 |
| Column totals | 4,826 | 2,232 | 7,058 |  | *p*-value | <0.0001 |
|  |  |  |  |  |  |  |
|  | **All others** | **Unknown** | **Row totals** |  | **Test values** |  |
| **Autoimmune** | 316 | 121 | 437 |  | Chi square stat | 3.34 |
| N/A | 4,510 | 2,111 | 6,621 |  | Critical value | 3.84 |
| Column totals | 4,826 | 2,232 | 7,058 |  | *p*-value | 0.07 |
|  |  |  |  |  |  |  |
|  | **All others** | **Unknown** | **Row totals** |  | **Test values** |  |
| **Depression** | 612 | 249 | 861 |  | Chi square stat | 3.32 |
| N/A | 4,214 | 1,983 | 6,197 |  | Critical value | 3.84 |
| Column totals | 4,826 | 2,232 | 7,058 |  | *p*-value | 0.07 |
|  |  |  |  |  |  |  |
|  | **All others** | **Unknown** | **Row totals** |  | **Test values** |  |
| **Sleep disorder** | 696 | 288 | 984 |  | Chi square stat | 2.93 |
| N/A | 4,130 | 1,944 | 6,074 |  | Critical value | 3.84 |
| Column totals | 4,826 | 2,232 | 7,058 |  | *p*-value | 0.09 |
|  |  |  |  |  |  |  |
|  | **All others** | **Unknown** | **Row totals** |  | **Test values** |  |
| **Coronary artery disease** | 630 | 294 | 924 |  | Chi square stat | 0.02 |
| N/A | 4,196 | 1,938 | 6,134 |  | Critical value | 3.84 |
| Column totals | 4,826 | 2,232 | 7,058 |  | *p*-value | 0.89 |
|  |  |  |  |  |  |  |
|  | **All others** | **Unknown** | **Row totals** |  | **Test values** |  |
| **Thymoma^2^** | 427 | 203 | 630 |  | Chi square stat | 0.11 |
| N/A | 4,399 | 2,029 | 6,428 |  | Critical value | 3.84 |
| Column totals | 4,826 | 2,232 | 7,058 |  | *p*-value | 0.73 |
|  |  |  |  |  |  |  |
|  | **All others** | **Unknown** | **Row totals** |  | **Test values** |  |
| **Obesity** | 833 | 367 | 1,200 |  | Chi square stat | 0.72 |
| N/A | 3,993 | 1,865 | 5,858 |  | Critical value | 3.84 |
| Column totals | 4,826 | 2,232 | 7,058 |  | *p*-value | 0.39 |
|  |  |  |  |  |  |  |
| **Exacerbation** | **All others** | **Unknown** | **Row totals** |  | **Test values** |  |
| Exacerbation at index Date | 578 | 256 | 834 |  | Chi square stat | 0.38 |
| N/A | 4,248 | 1,976 | 6,224 |  | Critical value | 3.84 |
| Column totals | 4,826 | 2,232 | 7,058 |  | *p*-value | 0.54 |
|  |  |  |  |  |  |  |
| **Inpatient diagnosis** | **All others** | **Unknown** | **Row totals** |  | **Test values** |  |
| Inpatient | 420 | 185 | 605 |  | Chi square stat | 0.33 |
| N/A | 4,406 | 2,047 | 6,453 |  | Critical value | 3.84 |
| Column totals | 4,826 | 2,232 | 7,058 |  | *p*-value | 0.56 |

¹ Includes commercial and Medicaid; commercial and Medicare; Medicare and Medicaid; and commercial, Medicare, and Medicaid.

^2^ Assessed within the 2-year study period after index date.

CCI, Charlson comorbidity index; GERD, gastroesophageal reflux disease; N/A, not applicable.

## Supplementary Table 3. Baseline demographics and characteristics of patients living with MG.

|  |  | **Racial/ethnic subgroups** | | | | |  |
| --- | --- | --- | --- | --- | --- | --- | --- |
|  | **Overall** | **Non- Hispanic**  **Caucasian** | **Non-Hispanic**  **African American** | **Hispanic** | **Asian** | **Unknown** | ***p*-value^3^** |
| *N* (%) | 7,058 | 4,052 (57) | 445 (6) | 235 (3) | 94 (1) | 2,232 (32) |  |
|  |  |  |  |  |  |  |  |
| Age, years |  |  |  |  |  |  |  |
| Mean (SD) | 61.93 (15.26) | 63.56 (14.7) | 52.72 (15.42) | 56.5 (16.91) | 56.43 (15.4) | 61.62 (15.22) | <0.0001* |
|  |  |  |  |  |  |  |  |
| Sex, *n* (%) |  |  |  |  |  |  |  |
| Female | 3,644 (52) | 2,015 (50) | 294 (66) | 142 (60) | 55 (59) | 1,138 (51) | <0.0001* |
| Male | 3,413 (48) | 2,036 (50) | 151 (34) | 93 (40) | 39 (41) | 1,094 (49) |  |
|  |  |  |  |  |  |  |  |
| Region, *n* (%) |  |  |  |  |  |  |  |
| Midwest | 2,629 (37) | 1,791 (44) | 187 (42) | 47 (20) | 28 (30) | 576 (26) | <0.0001* |
| Northeast | 1,694 (24) | 716 (18) | 87 (20) | 47 (20) | 28 (30) | 816 (37) |  |
| Other/Unknown | 292 (4) | 148 (4) | 14 (3) | 10 (4) | 2 (2) | 118 (5) |  |
| South | 1,830 (26) | 1,073 (26) | 150 (34) | 109 (46) | 17 (18) | 481 (22) |  |
| West | 613 (9) | 324 (8) | 7 (2) | 22 (9) | 19 (20) | 241 (11) |  |
|  |  |  |  |  |  |  |  |
| Insurance status, *n* (%) |  |  |  |  |  |  |  |
| Commercial | 3,322 (47) | 1,920 (47) | 209 (47) | 112 (48) | 52 (55) | 1,029 (46) | <0.0001* |
| Medicare | 2,349 (33) | 1,450 (36) | 93 (21) | 72 (31) | 24 (26) | 710 (32) |  |
| Medicaid | 365 (5) | 105 (3) | 74 (17) | 25 (11) | 9 (10) | 152 (7) |  |
| Multiple/unknown^1^ | 1,022 (14) | 577 (14) | 69 (16) | 26 (11) | 9 (10) | 341 (15) |  |
|  |  |  |  |  |  |  |  |
| CCI, mean (SD) | 1.33 (1.82) | 1.33 (1.76) | 1.25 (1.78) | 1.38 (2.02) | 1.13 (1.55) | 1.37 (1.91) | 0.71 |
| 0 | 3,082 (44) | 1,749 (43) | 212 (48) | 108 (46) | 46 (49) | 967 (43) |  |
| 1-2 | 2,708 (38) | 1,574 (39) | 155 (35) | 85 (36) | 34 (36) | 860 (39) |  |
| 3-4 | 827 (12) | 483 (12) | 51 (11) | 22 (9) | 9 (10) | 262 (12) |  |
| ≥5 | 441 (6) | 246 (6) | 27 (6) | 20 (9) | 5 (5) | 143 (6) |  |
|  |  |  |  |  |  |  |  |
| Baseline MG comorbidities, *n* (%) |  |  |  |  |  |  |  |
| Hypertension | 3,450 (49) | 2,027 (50) | 210 (47) | 116 (49) | 38 (40) | 1,059 (47) | 0.13 |
| Hyperlipidemia | 2,847 (40) | 1,697 (42) | 120 (27) | 105 (45) | 32 (34) | 893 (40) | <0.0001* |
| Diabetes | 1,866 (26) | 1,051 (26) | 116 (26) | 66 (28) | 21 (22) | 612 (27) | 0.59 |
| Thyroid-related disorders | 1,411 (20) | 853 (21) | 70 (16) | 43 (18) | 19 (20) | 426 (19) | 0.05 |
| GERD | 1,209 (17) | 735 (18) | 53 (12) | 51 (22) | 13 (14) | 357 (16) | <0.0001* |
| Anxiety | 827 (12) | 508 (13) | 49 (11) | 33 (14) | 11 (12) | 226 (10) | 0.05 |
| Autoimmune | 437 (6) | 264 (7) | 25 (6) | 18 (8) | 9 (10) | 121 (5) | 0.2 |
| Depression | 861 (12) | 522 (13) | 41 (9) | 37 (16) | 12 (13) | 249 (11) | 0.03* |
| Sleep disorder | 984 (14) | 596 (15) | 48 (11) | 38 (16) | 14 (15) | 288 (13) | 0.07 |
| Coronary artery disease | 924 (13) | 571 (14) | 26 (6) | 22 (9) | 11 (12) | 294 (13) | 0.47 |
| Other MG-associated conditions, *n* (%)^2^ |  |  |  |  |  |  |  |
| Thymoma | 630 (9) | 337 (8) | 50 (11) | 25 (11) | 15 (16) | 203 (9) | 0.02* |
|  |  |  |  |  |  |  |  |
| Baseline obesity/  overweight, *n* (%) | 1,200 (17) | 695 (17) | 81 (18) | 49 (21) | 8 (9) | 367 (16) | 0.08 |
|  |  |  |  |  |  |  |  |
| Exacerbation diagnosis at index, *n* (%) | 834 (12) | 474 (12) | 50 (11) | 40 (17) | 14 (15) | 256 (11) | 0.12 |
| Inpatient diagnosis,  *n* (%) | 605 (9) | 354 (9) | 40 (9) | 21 (9) | 5 (5) | 185 (8) | 0.78 |

¹ Includes commercial and Medicaid; commercial and Medicare; Medicare and Medicaid; and commercial, Medicare, and Medicaid.

^2^ Assessed within the 2-year study period after index date.

^3^ *p*-value was calculated via the t-test for continuous variables and chi-square test for categorical.

* *p*-value <0.05 was considered statistically significant.

CCI, Charlson comorbidity index; GERD, gastroesophageal reflux disease; MG, myasthenia gravis; SD, standard deviation.

## Supplementary Table 4. Treatment types.

|  |  | **Overall** | **Non- Hispanic**  **Caucasian** | **Non-Hispanic**  **African American** | **Hispanic** | **Asian** | **Unknown** |
| --- | --- | --- | --- | --- | --- | --- | --- |
| *N* (%) |  | 7,058 | 4,052 (57) | 445 (6) | 235 (3) | 94 (1) | 2,232 (32) |
| **First year** | | | | | | | |
| ACHE | *n* (%) | 4,129 (59) | 2,405 (59) | 215 (48) | 140 (60) | 63 (67) | 1,306 (59) |
| Glucocorticoids | *n* (%) | 3,210 (45) | 1,882 (46) | 201 (45) | 108 (46) | 46 (49) | 973 (44) |
| NSIST | *n* (%) | 1,260 (18) | 741 (18) | 71 (16) | 45 (19) | 21 (22) | 382 (17) |
| IVIG + SCIG | *n* (%) | 777 (11) | 465 (11) | 28 (6) | 28 (12) | 12 (13) | 244 (11) |
| PLEX | *n* (%) | 370 (5) | 227 (6) | 27 (6) | 7 (3) | 5 (5) | 104 (5) |
| Rituximab | *n* (%) | 61 (1) | 35 (1) | <5 (<3) | <5 (<3) | <5 (<3) | 18 (1) |
| Eculizumab | *n* (%) | 23 (0) | 13 (0) | - (0) | - (0) | - (0) | 10 (0) |
|  |  |  |  |  |  |  |  |
| **Thymectomy** | *n* (%) | 234 (3) | 125 (3) | 20 (4) | 9 (4) | 7 (7) | 73 (3) |
|  |  |  |  |  |  |  |  |
| **Second year** | | | | | | | |
| ACHE | *n* (%) | 3,094 (44) | 1,759 (43) | 195 (44) | 112 (48) | 44 (47) | 984 (44) |
| Glucocorticoids | *n* (%) | 2,717 (38) | 1,594 (39) | 182 (41) | 84 (36) | 28 (30) | 829 (37) |
| NSIST | *n* (%) | 1,329 (19) | 782 (19) | 76 (17) | 50 (21) | 23 (24) | 398 (18) |
| IVIG + SCIG | *n* (%) | 641 (9) | 370 (9) | 33 (7) | 27 (11) | 9 (10) | 202 (9) |
| PLEX | *n* (%) | 190 (3) | 116 (3) | 14 (3) | 8 (3) | <5 (<3) | 51 (2) |
| Rituximab | *n* (%) | 85 (1) | 52 (1) | <5 (<3) | 6 (3) | <5 (<3) | 24 (1) |
| Eculizumab | *n* (%) | 45 (1) | 29 (1) | - (0) | - (0) | <5 (<3) | 15 (1) |
|  |  |  |  |  |  |  |  |
| **Thymectomy** | *n* (%) | 48 (1) | 33 (1) | <5 (<3) | <5 (<3) | - (0) | 9 (0) |
|  |  |  |  |  |  |  |  |
| **0- to 90-day period** | | | | | | | |
| ACHE | *n* (%) | 3,456 (49) | 2,037 (50) | 179 (40) | 128 (54) | 51 (54) | 1,061 (48) |
| Glucocorticoids | *n* (%) | 2,022(29) | 1,189(29) | 123 (28) | 75 (32) | 29 (31) | 606 (27) |
| NSIST | *n* (%) | 637 (9) | 372 (9) | 35 (8) | 19 (8) | 14 (15) | 197 (9) |
| IVIG + SCIG | *n* (%) | 442 (6) | 271 (7) | 15 (3) | 11 (5) | 8 (9) | 137 (6) |
| PLEX | *n* (%) | 226 (3) | 137 (3) | 13 (3) | 5 (2) | <5 (<3) | 67 (3) |
| Rituximab | *n* (%) | 21 (0) | 12 (0) | - (0) | - (0) | <5 (<3) | 8 (0) |
| Eculizumab | *n* (%) | <5 (<3) | <5 (<3) | - (0) | - (0) | - (0) | <5 (<3) |
|  |  |  |  |  |  |  |  |
| **Thymectomy** | *n* (%) | 109 (2) | 55 (1) | 5 (1) | <5 (<3) | <5 (<3) | 44 (2) |

ACHE, acetylcholinesterase inhibitor; IVIG, intravenous immunoglobulin; SCIG, subcutaneous immunoglobulin; NSIST, nonsteroidal immunosuppressive treatment; PLEX, plasma exchange.

## Supplementary Table 5. Acute care outcomes.

|  |  | **Overall** | **Non- Hispanic**  **Caucasian** | **Non-Hispanic**  **African American** | **Hispanic** | **Asian** | **Unknown** |
| --- | --- | --- | --- | --- | --- | --- | --- |
| *N* (%) |  | 7,058 | 4,052 (57) | 445 (6) | 235 (3) | 94 (1) | 2,232 (32) |
| **First year (primary)** | | | | | | | |
| **Hospitalizations** | | | | | | | |
| MG-specific hospitalizations | *n* (%) | 925 (13) | 538 (13) | 48 (11) | 32 (14) | 13 (14) | 294 (13) |
| Hospital LOS | Mean (SD) | 6.93 (9.35) | 6.65 (8.61) | 7.41 (10.38) | 8.28 (8.3) | 8.88 (10.53) | 7.21 (10.58) |
|  | Median (range) | 4 (1-89) | 4 (1-89) | 4 (1-59) | 5 (1-30) | 4 (1-35) | 4 (1-83) |
|  |  |  |  |  |  |  |  |
| **ED visits** | | | | | | | |
| MG-specific ED visits | *n* (%) | 900 (13) | 507 (13) | 73 (16) | 33 (14) | 5 (5) | 282 (13) |
|  |  |  |  |  |  |  |  |
| **Outpatient visits** | | | | | | | |
| MG-specific outpatient visits | *n* (%) | 6,343 (90) | 3,669 (91) | 384 (86) | 197 (84) | 88 (94) | 2,005 (90) |
| MG-specific outpatient visits per patient | Mean (SD) | 4.6 (4.1) | 4.70 (4.15) | 3.62 (2.82) | 5.22 (4.79) | 4.49 (3.82) | 4.59 (4.13) |
|  | Median (range) | 3 (1-74) | 4 (1-74) | 3 (1-16) | 4 (1-36) | 3 (1-19) | 3 (1-44) |
|  |  |  |  |  |  |  |  |
| **Hospitalization-related events** | | | | | | | |
| Number of patients with a crisis event | MG-hospitalized cohort | 77 (8) | 43 (8) | 5 (10) | <5 (<3) | <5 (<3) | 25 (9) |
|  |  |  |  |  |  |  |  |
| **Exacerbations** | | | | | | | |
| Number of exacerbations | *n* (%) | 2,343 (33) | 1,353 (33) | 148 (33) | 86 (37) | 38 (40) | 718 (32) |
| Number of exacerbations at index date | *n* (%) of total exacerbations | 834 (36) | 474 (35) | 50 (34) | 40 (47) | 14 (37) | 256 (36) |
|  |  |  |  |  |  |  |  |
| **Thymectomy visits^1^** | | | | | | | |
| MG-specific thymectomy visits | *n* (%) | 234 (3) | 125 (3) | 20 (4) | 9 (4) | 7 (7) | 73 (3) |
| **Second year (exploratory)** | | | | | | | |
| **Hospitalizations** | | | | | | | |
| MG-specific hospitalizations | *n* (%) | 358 (5) | 203 (5) | 19 (4) | 23 (10) | <5 (<3) | 110 (5) |
| Hospital LOS | Mean (SD) | 6.99 (17.04) | 6.30 (9.95) | 15.07 (63.58) | 7.46 (9.81) | 1.25 (0.5) | 7.07 (9.81) |
|  | Median (range) | 3 (1-333) | 3 (1-83) | 2 (1-333) | 4 (1-36) | 1 (1-2) | 4 (1-69) |
|  |  |  |  |  |  |  |  |
| **ED visits** | | | | | | | |
| MG-specific ED visits | *n* (%) | 465 (7) | 258 (6) | 43 (10) | 25 (11) | 5 (5) | 134 (6) |
|  |  |  |  |  |  |  |  |
| **Outpatient visits** | | | | | | | |
| MG-specific outpatient visits | *n* (%) | 4,847 (69) | 2,803 (69) | 305 (69) | 154 (66) | 65 (69) | 1,520 (68) |
| MG-specific outpatient visits per patient | Mean (SD) | 3.42 (3.21) | 3.43 (3.17) | 3.00 (2.63) | 3.88 (3.6) | 2.95 (2.7) | 3.44 (3.37) |
|  | Median (range) | 2 (1-55) | 2 (1-40) | 2 (1-16) | 3 (1-26) | 2 (1-12) | 2 (1-55) |
|  |  |  |  |  |  |  |  |
| **Hospitalization-related events** |  |  |  |  |  |  |  |
| Number of patients with a crisis event | MG-hospitalized cohort | 18 (5) | 7 (3) | <5 (<3) | 0 (0) | 0 (0) | 10 (9) |
|  |  |  |  |  |  |  |  |
| **Exacerbations** | | | | | | | |
| Number of exacerbations | *n* (%) | 1,446 (20) | 811 (20) | 95 (21) | 60 (26) | 14 (15) | 466 (21) |
|  |  |  |  |  |  |  |  |
| **Thymectomy visits^1^** | | | | | | | |
| MG-specific thymectomy visits | *n* (%) | 48 (1) | 33 (1) | <5 (<3) | <5 (<3) | 0 (0) | 9 (0) |
|  |  |  |  |  |  |  |  |
| **Overall (first + second year)** | | | | | | | |
| **Hospitalizations** | | | | | | | |
| MG-specific hospitalizations | *n* (%) | 1,159 (16) | 674 (17) | 58 (13) | 51 (22) | 15 (16) | 361 (16) |
| Hospital LOS | Mean (SD) | 6.95 (11.96) | 6.55 (8.99) | 9.63 (35.08) | 7.97 (8.85) | 7.43 (9.91) | 7.17 (10.37) |
|  | Median (range) | 4 (1-333) | 3 (1-89) | 4 (1-333) | 4 (1-36) | 2 (1-35) | 4 (1-83) |
|  |  |  |  |  |  |  |  |
| **ED Visits** | | | | | | | |
| MG-specific ED visits | *n* (%) | 1,206 (17) | 683 (17) | 96 (22) | 50 (21) | 10 (11) | 367 (16) |
|  |  |  |  |  |  |  |  |
| **Outpatient visits** | | | | | | | |
| MG-specific outpatient visits | *n* (%) | 6,648 (94) | 3,829 (94) | 412 (93) | 212 (90) | 90 (96) | 2,105 (94) |
| MG-specific outpatient visits per patient | Mean (SD) | 6.89 (6.26) | 7.02 (6.24) | 5.60 (4.66) | 7.67 (7.4) | 6.52 (5.47) | 6.86 (4.13) |
|  | Median (range) | 5 (1-95) | 5 (1-95) | 4 (1-26) | 6 (1-46) | 5 (1-24) | 5 (1-91) |
|  |  |  |  |  |  |  |  |
| **Hospitalization-related events** | | | | | | | |
| Number of patients with a crisis event | MG-hospitalized cohort | 92 (8) | 50 (7) | 6 (1) | <5 (<3) | <5 (<3) | 32 (9) |
|  |  |  |  |  |  |  |  |
| **Exacerbations^1^** | | | | | | | |
| Number of exacerbations | *n* (%) | 2,782 (39) | 1,600 (39) | 173 (39) | 101 (43) | 43 (46) | 865 (39) |
| Number of exacerbations at index date patients | *n* (%) of total exacerbations | 834 (30) | 474 (30) | 50 (29) | 40 (40) | 14 (33) | 256 (30) |
| **Thymectomy visits^2^** | | | | | | | |
| MG-specific Thymectomy visits | *n* (%) | 282 (4) | 158 (4) | 24 (5) | 11 (5) | 7 (7) | 82 (4) |

ED; emergency department; LOS, length of stay; MG, myasthenia gravis, SD, standard deviation.

^1^ Identified based on the basis of diagnosis codes (358.01 and G70.01). Additionally, patients having only exacerbation codes throughout the observation period were not considered as part of this population as it is most likely a coding error.

^2^ Identified by checking for the presence of a thymectomy procedure during any MG-specific inpatient visit.

## Supplementary Table 6. Odds of MG-related ED visit: Sensitivity analysis using CCI as a dichotomous variable.

|  | **Year 1** | | **Year 2** | |
| --- | --- | --- | --- | --- |
| **Covariates** | **OR (95% CI)** | **p-value** | **OR (95% CI)** | **p-value** |
| Sex (Ref: Male) |  |  |  |  |
| Female | 0.93 (0.8-1.09) | 0.38 | 1.11 (0.89-1.37) | 0.35 |
| Age | 0.99 (0.98-0.99) | <0.001 | 0.98 (0.97-0.99) | <0.001 |
| Race (Ref: Caucasian) |  |  |  |  |
| African American | 1.38 (1.03-1.84) | 0.02 | 1.23 (0.85-1.78) | 0.27 |
| Hispanic | 0.89 (0.59-1.34) | 0.58 | 1.24 (0.77-2) | 0.37 |
| Asian | 0.35 (0.14-0.87) | 0.02 | 0.69 (0.27-1.74) | 0.43 |
| Unknown | 0.99 (0.84-1.17) | 0.91 | 0.85 (0.68-1.07) | 0.17 |
| Insurance (Ref: Commercial) |  |  |  |  |
| Medicare | 1 (0.82-1.22) | 0.98 | 1.21 (0.92-1.6) | 0.18 |
| Medicaid | 1.93 (1.45-2.57) | <0.001 | 2.96 (2.13-4.13) | <0.001 |
| Multiple/unknown | 1.15 (0.92-1.43) | 0.23 | 1.58 (1.19-2.09) | <0.001 |
| Comorbidities (Ref: CCI <2) |  |  |  |  |
| CCI ≥2 | 1.22 (1.01-1.46) | 0.04 | 1.21 (0.94-1.56) | 0.14 |
| Obese/overweight | 1.15 (0.95-1.39) | 0.15 | 1.04 (0.79-1.36) | 0.80 |
| Exacerbation at index | 2.14 (1.78-2.59) | <0.001 | 1.24 (0.94-1.62) | 0.13 |
| Outpatient visits | 1.14 (1.12-1.16) | <0.001 | 1.09 (1.08-1.1) | <0.001 |

## Supplementary Table 7. Odds of MG-related hospitalization: Sensitivity analysis using CCI as a dichotomous variable.

|  | **Year 1** | | **Year 2** | |
| --- | --- | --- | --- | --- |
| **Covariates** | **OR (95% CI)** | **p-value** | **OR (95% CI)** | **p-value** |
| Sex (Ref: Male) |  |  |  |  |
| Female | 0.95 (0.81-1.12) | 0.54 | 1 (0.78-1.27) | 0.97 |
| Age | 0.99 (0.98-0.99) | <0.001 | 0.99 (0.98-1.00) | 0.03 |
| Race (Ref: Caucasian) |  |  |  |  |
| African American | 0.94 (0.66-1.33) | 0.71 | 0.9 (0.54-1.5) | 0.68 |
| Hispanic | 0.76 (0.48-1.18) | 0.22 | 1.71 (1.03-2.82) | 0.04 |
| Asian | 0.94 (0.48-1.83) | 0.85 | 0.68 (0.21-2.19) | 0.52 |
| Unknown | 0.99 (0.83-1.17) | 0.89 | 0.99 (0.77-1.27) | 0.94 |
| Insurance (Ref: Commercial) |  |  |  |  |
| Medicare | 1.33 (1.09-1.62) | 0.01 | 1.09 (0.81-1.48) | 0.58 |
| Medicaid | 0.75 (0.51-1.11) | 0.16 | 1.2 (0.74-1.94) | 0.47 |
| Multiple/unknown | 1.13 (0.89-1.43) | 0.31 | 1.34 (0.97-1.86) | 0.08 |
| Comorbidities (Ref: CCI <2) |  |  |  |  |
| CCI ≥2 | 1.58 (1.31-1.9) | <0.001 | 1.14 (0.86-1.51) | 0.38 |
| Obese/overweight | 1.23 (1.01-1.49) | 0.04 | 1.43 (1.08-1.9) | 0.01 |
| Exacerbation at index | 2.71 (2.26-3.26) | <0.001 | 1.46 (1.08-1.96) | 0.01 |
| Outpatient visits | 1.15 (1.13-1.16) | <0.001 | 1.08 (1.07-1.1) | <0.001 |
